# Empagliflozin ameliorates symptoms of diabetes and renal tubular dysfunction in a rat model of diabetes with enlarged kidney (DEK)

**DOI:** 10.1371/journal.pone.0251135

**Published:** 2021-05-04

**Authors:** Ayaka Domon, Kentaro Katayama, Touko Sato, Yuki Tochigi, Hiroyuki Tazaki, Hiroetsu Suzuki

**Affiliations:** 1 Laboratory of Veterinary Physiology, Nippon Veterinary and Life Science University, Tokyo, Japan; 2 Laboratory of Biomolecular Chemistry, Nippon Veterinary and Life Science University, Tokyo, Japan; Max Delbruck Centrum fur Molekulare Medizin Berlin Buch, GERMANY

## Abstract

**Background:**

Sodium-glucose cotransporter 2 (SGLT2) inhibitors are widely used to reduce hyperglycemia. The present study investigated the effects of a SGLT2 inhibitor, empagliflozin, on hyperglycemia in a novel rat model of non-obesity type 2 diabetes with enlarged kidney (DEK).

**Methods:**

Male DEK rats with non-fasting blood glucose concentrations ≤300 mg/dl and >300 mg/dl were classified as nondiabetic and diabetic, respectively. Groups of nondiabetic (control) and diabetic (DM-cont) rats were fed standard chow for 12 weeks, whereas another group of diabetic (DM-empa) rats was fed standard chow containing empagliflozin (300 mg/kg/day) for 12 weeks. Blood glucose, body weight, glucose tolerance, food and water intake, urinary volume, plasma and urinary biochemical parameters, and bone mineral density were measured, and their kidneys and pancreas histologically analyzed.

**Results:**

Treatment with empagliflozin reduced blood glucose concentration and food intake in diabetic rats, but inhibited loss of adeps renis and led to body weight gain. Empagliflozin attenuated polyuria and polydipsia but increased plasma concentrations of total cholesterol, sodium and total protein toward normal level. Empagliflozin also significantly reduced urinary excretion of proteins and electrolytes and restored bone mineral density and plasma concentrations of valine and isoleucine to normal levels. Moreover, dilation of renal tubules and kidney enlargement were not attenuated in the DM-empa group.

**Conclusion:**

The response of DEK rats to empagliflozin differed from that of other diabetic animal models, suggesting that DEK rats have unique characters for studying and evaluating the multiple biological effects of SGLT2 inhibitors. These findings also indicted that empagliflozin could ameliorate systemic metabolism and improve renal tubule function in diabetic condition.

## Introduction

The worldwide prevalence of diabetes mellitus (DM) continues to increase, with the number of patients estimated to reach 700 million by 2045 [1]. About 90–95% of these patients have type 2 DM, which is characterized by progressive reduction of insulin secretion with or without insulin resistance [2] and is a major risk factor for renal and cardiovascular diseases [3]. Type 2 DM is a leading cause of end-stage renal failure (ESRD), with diabetic nephropathy, also called diabetic kidney diseases, being responsible for ESRD in 20–40% of these patients [4]. Patients with ESRD require dialysis or kidney transplantation to maintain normal kidney function. These procedures place social and economic burdens on societies and individuals, as well as affecting patient quality of life [5]. Efforts are therefore needed to prevention or ameliorate DM and diabetic nephropathy [6].

Sodium-glucose cotransporter 2 (SGLT2) inhibitors are a novel class of anti-diabetic drugs that act independent of endogenous insulin [7]. SGLT2 inhibitors reduce blood glucose concentrations by inhibiting glucose reabsorption at proximal renal tubules [7]. SGLT2 inhibitors also have beneficial effects on renal and cardiovascular complication of DM [8–10], as well as improving metabolism [11], all of which are partly independent of their glucose lowering effects. Furthermore, SGLT2 inhibitors improve renal outcome in non-DM patients with chronic kidney diseases [12]. However, the effects of SGLT2 inhibitors, including renal outcomes, vary among patients [13]. These variations likely reflect differences in multiple factors, including genetic predisposition, metabolic status, hemodynamics and lifestyle.

We recently established a novel rat strain of non-obesity type 2 DM with enlarged kidney (DEK) from LEA.PET-*pet* congenic strain. In the DEK strain, about 50 percent of males show diabetes by 30 weeks of age. These diabetic rats showed progressive reduction in plasma insulin concentration due to loss of pancreatic β cells, increased renal parenchyma and marked enlargement of the kidneys with dilation of renal tubules [14]. DEK rats also showed reductions in plasma concentrations of total cholesterol (TCHO), total protein and albumin [14]. These histological and metabolic features are unique to DEK rats, distinguishing this model from other animal models of diabetes and suggesting that DEK rats could be useful for studying the pharmacological effects of anti-diabetic medications on kidneys and metabolism. The present study therefore evaluated the effects of the SGLT2 inhibitor, empagliflozin on the kidneys and systemic metabolism of DEK rats.

## Materials and methods

### Animals

Blood samples were obtained from the tail veins of non-fasting male DEK rats (total n = 33), and glucose concentrations were measured with GLUCOCARD PlusCare (Arkray Inc., Kyoto, Japan). Male DEK rats with non-fasting blood glucose concentrations ≤300 mg/dl and >300 mg/dl were classified as nondiabetic and diabetic, respectively [14]. All rats were maintained under conventional conditions, including a 14:10 h light: dark cycle, a room temperature of 20±2°C and a relative humidity of 50±10%. All animal studies were approved by the Animal Care and Use Committee of Nippon Veterinary and Life Science University, and were performed in accordance with the Guidelines of the Animal Care and Use Committee of Nippon Veterinary and Life Science University.

Male rats with blood glucose concentrations >300 mg/dl at age 15–20 weeks in DEK strain were randomly divided into an empagliflozin diet group (DM-empa) (n = 5) and a standard diet group (DM-cont) (n = 16) within 2–3 weeks after developing DM. Male rats with the non-DM (blood glucose level was still lower than 300 mg/dl at 30 weeks of age) in DEK strain were defined as control (n = 12). Control and DM-cont groups had *ad libitum* access to water and standard diet (CR-LPF, Oriental Yeast Co., Ltd., Tokyo. Japan) for 12 weeks, whereas rats in the DM-empa group had *ad libitum* access to water and a standard diet plus empagliflozin (300 mg/kg; Eli Lilly Japan; Kobe, Japan). The dose of empagliflozin was designed with reference to previous reports [15, 16]. Non-fasting blood glucose concentrations, body weight and food intake were measured weekly. All rats were sacrificed after 12 weeks, and their kidney, pancreases and blood samples were collected. We set humane endpoints for any animals who decreased 25% of body weight or showed symptoms associated to severely ill. However, there was no rats showing such severe reduction of body weight and ill condition in this study.

### Oral Glucose Tolerance Test (OGTT)

Oral glucose tolerance tests (OGTT) were performed the day after the 12 week treatment period, as described [14]. Briefly, the rats were fasted for 16 h and administered 2g/kg body weight (BW) of glucose orally. Blood was obtained from the tail vein, and glucose concentrations were measured 0, 30, 60, 120, 180, and 240 min after glucose loading. Blood samples were also collected from the jugular vein under isoflurane anesthesia controlled by small-animal anesthetizer (TK-7, Biomachinery, Chiba, Japan) at 0, 30 and 60 min after glucose loading, mixed with heparin and centrifuged for 15 min at 4°C to obtain plasma. Plasma insulin concentrations were measured using an LBIS Rat Insulin ELISA Kit (RTU) (Shibayagi, Gunma, Japan).

### Measurement of renal parameters

During week 12, the rats were housed individually in metabolic cages for 24 h, and urine volume and drinking water volume were measured [14]. Urinary concentrations of creatinine (Cre), urea nitrogen (UN), calcium (Ca), Na-K-Cl and glucose (Glu) were measured with Dri-Chem 3500V (FUJIFILM, Tokyo, Japan). Urinary protein concentrations were measured using Protein Assay Rapid Kit Wako II (FUJIFILM Wako Pure Chemical Corporation, Tokyo, Japan). Blood was collected from the caudal vena cava under isoflurane anesthesia, and plasma concentrations of UN, albumin (Alb), total protein (TP), total cholesterol (Tcho), Ca and Na-K-Cl were measured using Dri-Chem 3500V. Plasma concentration of Cre was measured using FUJIFILM VET Systems Co.,Ltd. (Tokyo, Japan). Creatinine (Ccre), glucose (Cglu), calcium (Cca), sodium (Can), potassium (Ck), and chlorine (Ccl) clearances were calculated using the formula:

Clearance (Cx) (ml/min/kg) = [urine concentration (mg/dl or mEq/l) × urine volume (ml/min)] / plasma concentration (mg/dl or mEq/l) / body weight (kg).

Fractional excretion of glucose (FEglu), calcium (FEca), sodium (FEna), potassium (FEk), and chlorine (FEcl) were calculated using the formula:

Fraction excretion (FEx) (%) = Cx (ml/min/kg) / Ccre (ml/min/kg) × 100.

The urinary concentration of liver fatty-acid-binding (L-FABP) was measured using Mouse/Rat FABP1/L-FABP kit (R&D Systems, Inc., MN, USA).

### Histology, immunofluorescence and calculation of insulin positive area

Animals were euthanized with overdose of pentobarbital sodium after 12 week treatment period. The kidneys and pancreas were removed from each euthanatized rat and weighed. Tissue samples were fixed with 4% paraformaldehyde in phosphate-buffered saline (PBS), embedded in paraffin following standard procedures and sectioned at 1 μm or 4 μm thickness for periodic acid-Schiff (PAS) and immunofluorescence staining. Renal tubular dilation was scored as described previously [17]. Ten random non-overlapping 40 x magnified fields of the renal cortex and of the juxtamedullary area were scored. Tubular dilation was scored on a scale of 0–3, with 0, 1, 2, and 3 indicating 0, <5, 5–10, and >10 dilated tubules per field, respectively. Pancreas sections were blocked with 3% bovine serum albumin (BSA) in PBS and incubated overnight at 4°C with guinea pig anti-insulin polyclonal antibody (1: 100, Abcam K.K., Tokyo, Japan) or mouse anti-glucagon polyclonal antibody (1: 200, Abcam K.K.). The sections were rinsed with PBS and incubated for one hour at room temperature with Alexa Fluor 488 conjugated goat anti-guinea pig IgG antibody (1: 1000, Life Technologies, Carlsbad, CA) or Alexa Fluor 568 conjugated donkey anti-mouse IgG antibody (1: 1000, Life Technologies). After rinsing in PBS, the sections were mounted with ProLong Gold Antifade Reagent with DAPI (Life Technologies, Carlsbad, CA). Images were acquired with a Biozero BZ-X800 all-in-one fluorescence microscope, (KEYENCE, Tokyo, Japan). Islet areas and insulin-positive areas were measured in >10 islet sections per rat using Image J software, and the percentage of insulin-positive area per islet area was calculated.

### Bone densitometry analysis of femurs

Femurs were excised from four euthanatized non-DM control, three DM-cont, and three DM-empa rats after 12 week treatment period and scanned with a micro-CT system (Latheta LCT-100, Aloka, Tokyo, Japan). Bone mineral density (BMD, mg/cm^3^) and bone mineral content (BMC, mg) were calculated using Latheta software.

### Gas chromatography-mass spectrometry (GC-MS) analysis of plasma amino acids

Plasma samples were obtained from 30-week-old control rats and from 27–32 week-old DM-cont and DM-empa rats after the 12-week treatment period. The plasma samples were treated with Phenomenex EZ:faast AA analysis kit (Phenomenex, Torrance, CA), with norvalin used as the internal standards [18]. GCMS-QP2010Plus and GCMS solution software (Shimadzu, Kyoto, Japan) were used for GC-MS analysis [19].

### Data processing of plasma amino acids

Partial Least Squares Discriminant Analysis (PLS-DA) was performed using SIMCA-P software (version 13.0.3, Umetrics, Umeda, Sweden). Multiple comparisons were performed using IBM SPSS Statistics 19 Documentation (version 19, IBM, Armonk, NY, USA).

### Statistical analysis

Results, presented as mean ± standard error (SE), were compared in two groups by two-tailed Student’s *t*-tests and in multiple groups by Tukey-Kramer tests. A *p* value < 0.05 was considered statistically significant.

## Results

### General conditions associated with diabetes

**Fig 1:**
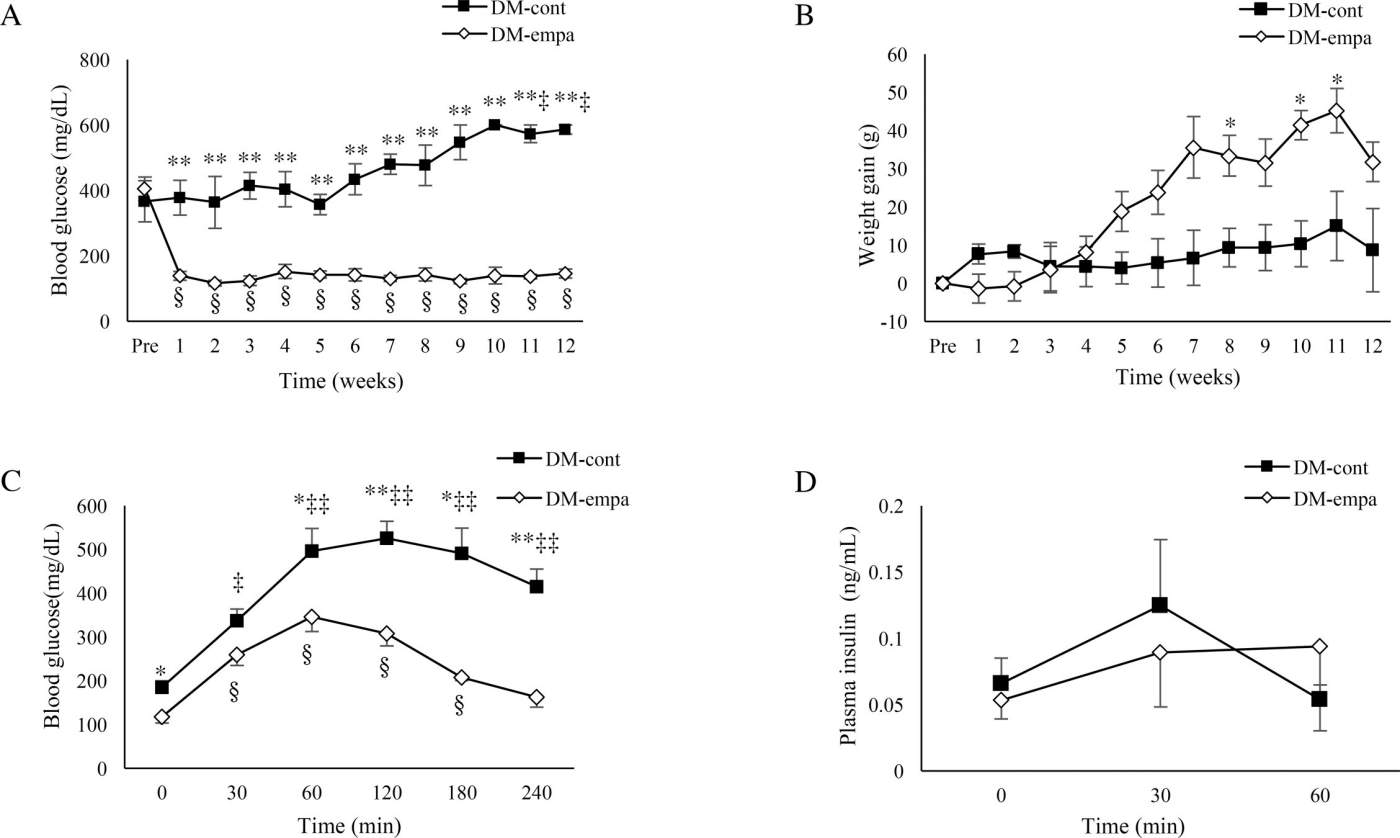
Effects of empagliflozin on blood glucose, body weight gain, and response to glucose loading. Blood glucose levels (A) and the gain of body weight (B) in DM-cont (n = 3) and DM-empa (n = 5) rats over 12 weeks. (C) Blood glucose levels after glucose loading in DM-cont (n = 5) and DM-empa (n = 5) rats. (D) Plasma insulin levels after glucose loading in DM-cont (n = 4) and DM-empa (n = 5) rats. * *p*<0.05, ** *p*<0.01 for comparisons between the DM-cont and DM-empa groups. ‡ *p*<0.05, ‡‡ *p*<0.01 for comparisons with pretreatment levels in the DM-cont group, §*p*<0.01 for comparisons with pretreatment levels in the DM-empa group.

Weekly measurements of food intake showed that the estimated average daily food intake was slightly higher in the DM-cont than in the DM-empa group (36.9±1.5 g vs. 34.3±1.0 g). The estimated daily dose of empagliflozin in the DM-empa group was 23.7±0.7 mg/kg body weight (BW). Empagliflozin dramatically reduced blood glucose concentration the day after starting treatment, from 404.6±36.3 mg/dl to 143.8±14.6 mg/dl), with blood glucose in the DM-empa group being significantly lower than in the DM-cont group during the 12-week treatment period (Fig 1A). This level in DM-empa was resemble for normal control with normal diet (147.0±1.2 mg/dl) [14]. BW gradually increased with age in the DM-empa group, but not in the DM-cont group (Fig 1B), with the final BW after 12 weeks being 444.6±18.0 g in the DM-empa and 372.3±26.4 g in the DM-cont group. At same age, the body weight of control was approximately 533.4±9.8 g [14]. Consistent with body weight gain, adeps renis (1.4±0.2 g) remained in the DM-empa but not in the DM-cont group.

**Fig 2:**
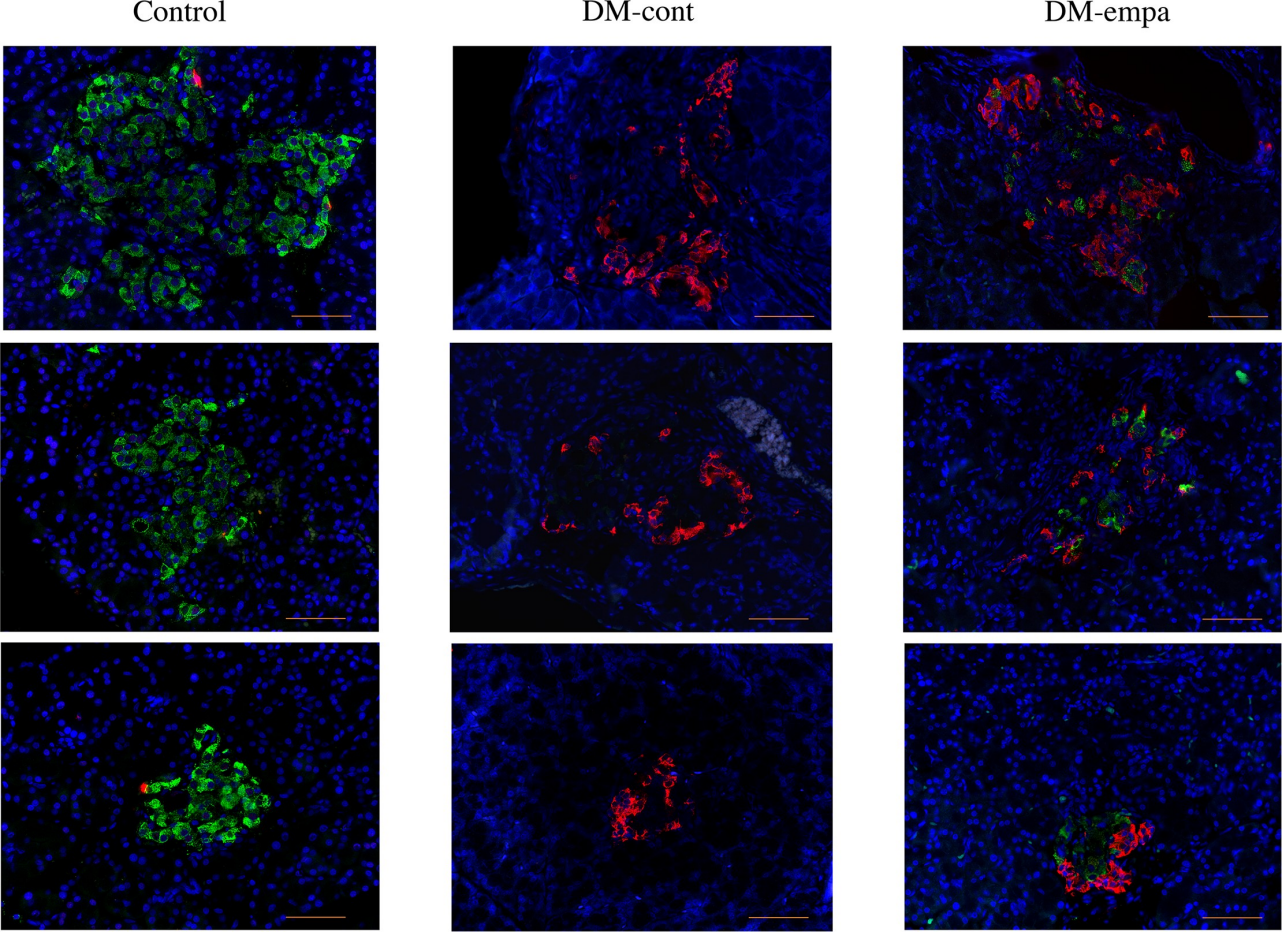
Assessment of insulin and glucagon producing cells after empagliflozin treatment. Immunologic detection of insulin (green) and glucagon (red) in pancreatic islets of rats in the control, DM-cont and DM-empa groups after 12 week treatment period. Scale bar: 50 μm.

Affected DEK rats were previously shown to exhibit severe glucose intolerance, no insulin secretion in response to glucose loading, and loss of β cells [14]. We examined whether empagliflozin improved glucose tolerance and insulin secretion and prevented loss of β cells. Blood glucose level after 16 hours of fasting was significantly lower in the DM-empa than in the DM-cont group, as was blood glucose level 60–240 min after glucose loading (Fig 1C). The time-related change of blood glucose in OGTT of DM-empa was almost resemble to that in non-diabetic control rats of DEK strain [14]. However, glucose induced-insulin secretion did not occur in either group (Fig 1D). Immunofluorescent staining of insulin in pancreatic islets showed no or faint insulin signals in the DM-cont group and almost 100% positive signals in control rats, whereas a weak insulin signal, comprising 24.2±3.5% per islet area was observed in three rats in the DM-empa group (Fig 2).

### Metabolic and renal parameters and bone densitometry

**Fig 3:**
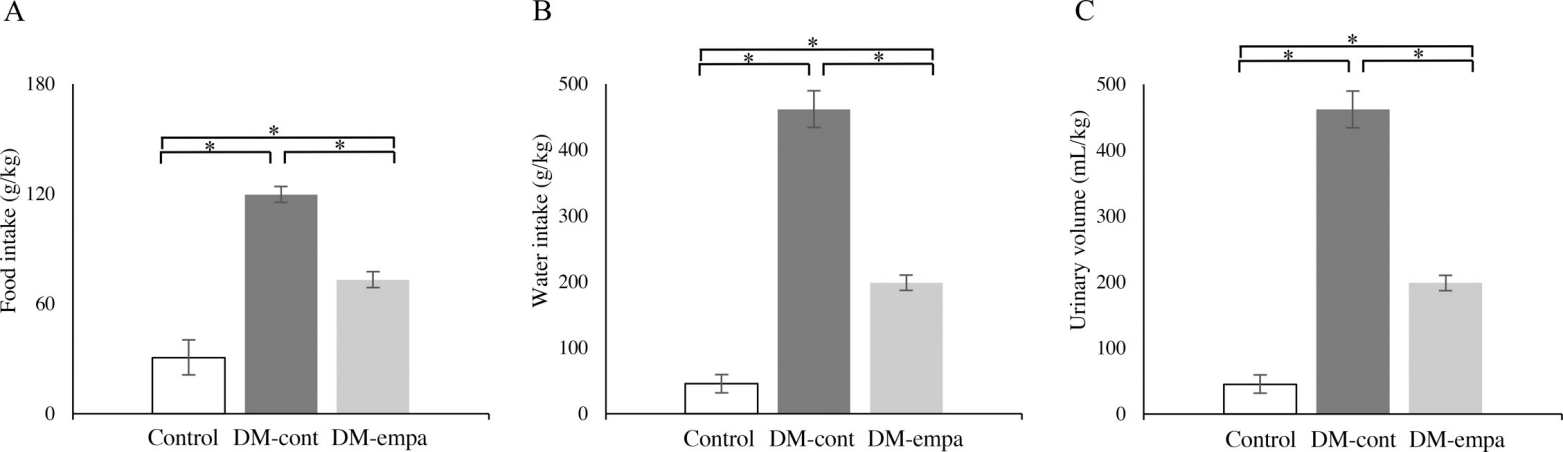
Effects of empagliflozin on food and water intake and urinary volume. Food intake (A), water intake (B) and urinary volume (C) over 24 h of rats in the control (n = 3), DM-cont (n = 3) and DM-empa (n = 3) groups during week 12. All data were expressed as relative to body weight. * *p*<0.05.

Measurements of metabolic parameters after 12 week treatment period showed that food intake was significantly lower in the DM-empa than in the DM-cont group in both relative (Fig 3A) and absolute (S1 Fig) values. In addition, water intake (Fig 3B, S1 Fig) and urine volume (Fig 3C, S1 Fig) were about 50% lower in the DM-empa than in the DM-cont group.

At the end of the 12-week treatment period, plasma Tcho, Cre, Alb, TP and Na concentrations were significantly lower in the DM-cont than in the control group, with most of these reductions in the DM-cont group showing partial recovery in the DM-empa group. In particular, plasma Tcho, TP, and Na concentrations were significantly higher in the DM-empa than in the DM-cont group, although all of these concentrations were lower in the DM-empa than in the control group. Plasma Alb and Cre levels were also higher in the DM-empa than in the DM-cont group, but these differences were not statistically significant (Table 1). Urinary excretions of glucose, UN, Ca, Na, K, Cl, and protein were significantly higher in the DM-cont than in the control group, with most of these increases alleviated in the DM-empa group. In particular, urinary excretions of Glu, UN, Na, K, and Cl were significantly lower in the DM-empa than in the DM-cont group, although all of these excretions were significantly higher in the DM-empa than in the control group. Increased urinary excretions of Ca and protein were also alleviated in the DM-empa group, with no significant difference between the DM-empa and control groups (Table 2). Except for Ccre, which did not differ significantly among the three groups, all clearances and fraction excretions examined were significantly higher in the DM-cont than in the control group (Table 3). Moreover, except for Cglu and FEglu, which remained higher in the DM-empa group, most of those increases in the DM-cont group were alleviated in the DM-empa group. In particular, Cca, Cna, and Ccl were significantly lower in the DM-empa than in the DM-cont group, but remained higher in the DM-empa than in the control group (Table 3).

**Table 1:** Comparison of plasma biochemical parameters between control, DM-cont, and DM-empa.

	Control (n = 3)	DM-cont (n = 3)	DM-empa (n = 5)
Glu (mg/dL)	113.7±9.3	573.0±27.0*	133.4±6.9#
Tcho (mg/dL)	94.3±4.4	62.0±3.1*	79.0±4.0#
Cre (mg/dL)	0.42±0.03	0.29±0.01*	0.30±0.02*
BUN (mg/dL)	16.2±2.3	21.1±0.5	22.1±3.1
Ca (mg/dL)	8.6±0.2	8.4±0.3	8.3±0.2
Na (mEq/L)	144.7±0.3	137.3±1.5*	141.0±0.5*#
K (mEq/L)	7.5±0.6	5.8±1.9	4.5±0.3
Cl (mEq/L)	100.3±0.3	98.7±0.3	100.8±1.0
Alb (g/dL)	3.8±0.1	2.6±0.1*	3.1±0.2*
TP (g/dL)	5.7±0.1	4.0±0.03*	4.4±0.1*#

Biochemical parameters in plasma after 12 weeks. Data are represented as mean ± SE.

* *p*<0.05 vs control.

# *p*<0.05 vs DM-cont.

**Table 2:** Comparison of 24 hours-urinary excretion relative to body weight between control, DM-cont, and DM-empa.

	Control (n = 3)	DM-cont (n = 3)	DM-empa (n = 5)
Glu (mg/kg)	5.09±0.94	39482.49±2862.57*	23864.64±2292.62*#
Cre (mg/kg)	58.17±8.20	56.71±1.70	56.45± 2.98
UN (mg/kg)	370.67±90.93	1303.43± 45.38*	907.90±52.99 *#
Ca (mg/kg)	3.73±0.39	35.68±3.06*	19.75±5.90
Na (mEq/kg)	2.96±0.77	11.22± 0.74*	7.45± 0.80*#
K (mEq/kg)	4.99±1.17	18.51± 1.25*	11.96± 0.97*#
Cl (mEq/kg)	4.14±1.00	13.89± 0.48*	9.79± 0.89*#
Protein (mg/kg)	16.66±3.54	163.55± 44.55*	78.81± 9.32

Twenty-four hour urinary excretion of Glu, Cre, UN, Ca, Na-K-Cl, and protein after 12 weeks. Data are represented as mean ± SE.

* *p*<0.05 vs control.

# *p*<0.05 vs DM-cont.

**Table 3:** Comparisons of clearance (mL/min/kg) and fractional excretion (%) of glucose, creatinine, major electrolytes between control, DM-cont, and DM-empa.

	Control (n = 3)	DM-cont (n = 3)	DM-empa (n = 5)
Cglu (× 10^2^)	0.30±0.02	477.30±12.82*	1252.02±126.93*#
Ccre	9.61±0.22	13.46±0.72	13.32±1.54
Cca (× 10^2^)	3.01±0.34	29.33±1.91*	16.18±4.51*#
Cna (× 10^2^)	1.34±0.12	5.70±0.44*	3.67±0.39*#
Ck	0.45±0.05	2.58±0.54*	1.88±0.20*
Ccl (× 10^2^)	2.74±0.22	9.78±0.35*	6.74±0.58*#
FEglu	0.03±0.002	35.62±1.71*	99.53±15.31*#
FEca	0.31±0.03	2.20±0.21*	1.27±0.34
FEna	0.14±0.01	0.43±0.05*	0.29±0.04*
FEk	4.66±0.45	19.05±3.85*	14.79±2.11*
FEcl	0.29±0.03	0.73±0.06*	0.53±0.06*

Clearance and fractional excretion of creatinine, sodium, and glucose after 12 weeks Data are represented as mean ± SE.

* *p*<0.05 vs control.

# *p*<0.05 vs DM-cont.

Femoral BMD was significantly lower in the DM-cont than in the control group, with empagliflozin found to restore femoral BMD in the DM-empa group (Table 4). BMC tended to be higher in the DM-empa than in the DM-cont group, but the difference was not statistically significant (Table 4).

**Table 4:** Comparison of bone densitometry between control, DM-cont, and DM-empa.

	Control (n = 4)	DM-cont (n = 3)	DM-empa (n = 3)
tBMD (mg/cm^3^)	868.1±6.9	760.6±22.2*	855.2±2.3#
cBMD (mg/cm^3^)	1236.5±5.1	1206.2±8.6*	1233.7±0.008#
tbBMD (mg/cm^3^)	445.9±10.1	375.9±12.5*	430.8±2.4#
tBMC (mg)	706.2±18.6	525.2±50.2*	577.1±41.8

Bone densitometric analysis of femurs in rats after 12 weeks. Abbreviations: tBMD, total bone mineral density; cBMD, cortical bone mineral density; tbBMD, trabecular bone mineral density; tBMC, total bone mineral content. Data are represented as mean ± SE.

* *p*<0.05 vs control.

# *p*<0.05 vs DM-cont.

### Pathology of the kidneys

**Fig 4:**
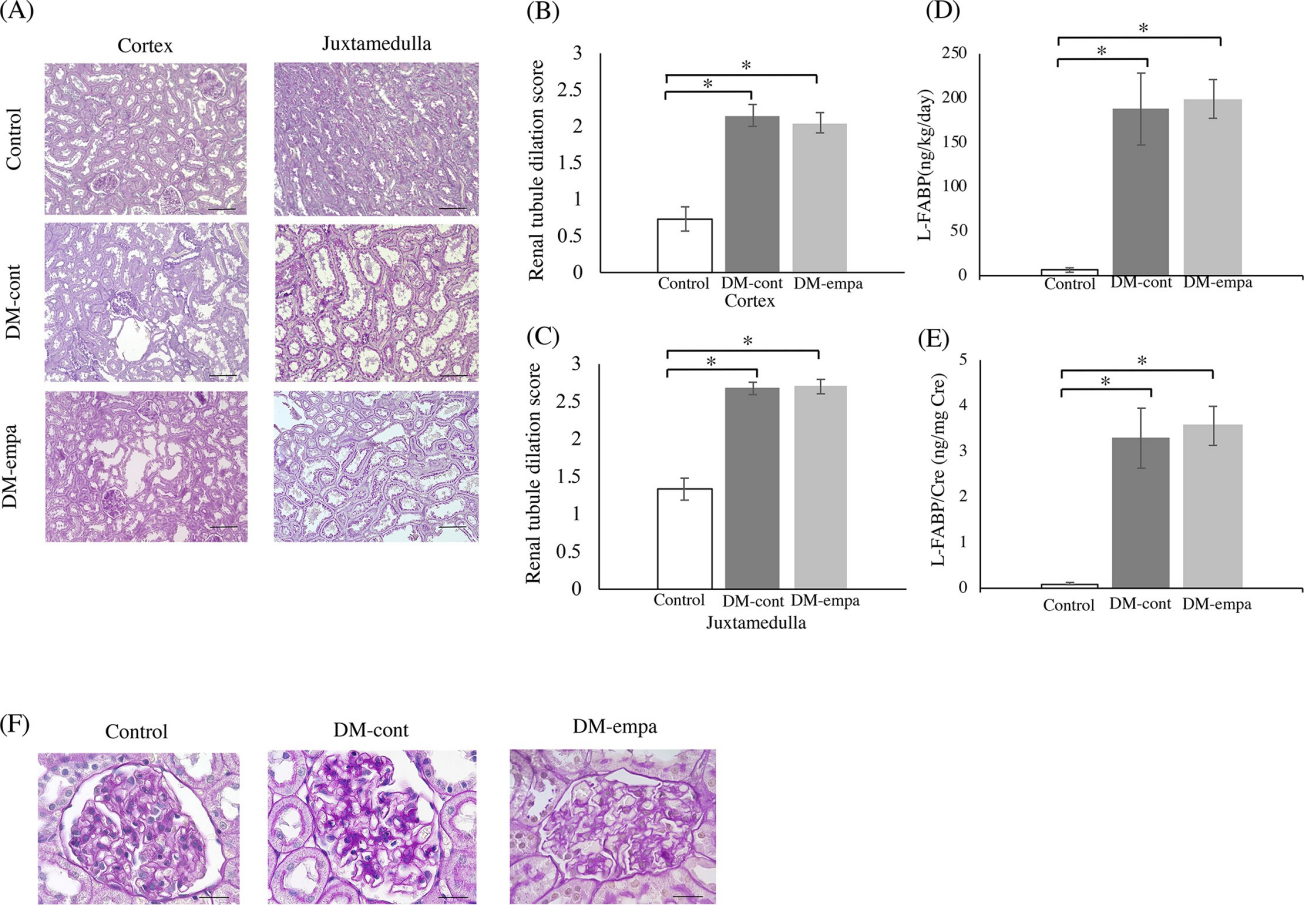
Assessment of renal histology and renal tubule dilation scores and urinary excretion of L-FABP after empagliflozin treatment. (A) Histology of the renal cortex and juxtamedulla. Sections were stained with PAS stain. Scale bar: 100μm. Renal tubule dilation scores of the cortex (B) and juxtamedulla (C). Urinary excretion of L-FABP for 24 hours (D, relative to body weight; E, relative to creatinine excretion). * *p*<0.05 vs Control. (F) Typical glomerular structure of control, DM-cont and DM-empa. Sections were stained with PAS stain. Scale bar: 20μm.

Affected DEK rats exhibited progressive kidney enlargement with dilated tubules [14]. Although empagliflozin treatment dramatically reduced blood glucose levels, it did not reduce kidney weight, which was similar in the DM-empa and DM-cont groups (2.35±0.08 g vs. 2.24±0.08 g). Histologic examination showed that severe dilation was prominent in renal tubules of the juxtamedullary regions in both DM-cont and DM-empa rats (Fig 4A). Empagliflozin did not attenuate the dilation in renal tubules (Fig 4B and 4C). In line with this, urinary excretion of L-FABP, a biomarker of renal tubular injury [20], was higher in DM-cont and DM-empa compared to Control, and there was no difference between DM-cont and DM-empa (Fig 4D and 4E). As previously reported [14], there were no typical glomerular lesion such as hypertrophy in Control, DM-cont, and DM-empa (Fig 4F).

### GC-MS analysis of plasma amino acids

Metabolic profiling using PLS-DA multivariate analysis showed differences in amino acid status among the control, DM-cont and DM-empa groups (S2 Fig). The loading plot corresponded to a score plot consisted of 24 amino acids (S2 Fig). The variable influences on projection (VIP) parameters are shown in S2 Fig. Amino acids colored red had VIP >1 and were considered to have a relatively high impact for separating these groups. Moreover, statistical analysis of amino acids showed a VIP >1.

Plasma sarcosine tended to be higher in the DM-cont than in the control group, with empagliflozin further increasing plasma sarcosine. In contrast, plasma tryptophan tended to be lower in the DM-cont than in the control group, with empagliflozin further decreasing plasma tryptophan (Table 5). Plasma glycine levels were similar in the DM-cont and control groups, but were significantly higher in the DM-empa than in the other two groups. Glutamine and ornithine concentrations were significantly lower in the DM-cont than in the control group and were not restored by empagliflozin treatment. Compared with the control group, plasma α-aminobutyric acid was significantly lower in the DM-cont group, with empagliflozin further reducing its concentration. Plasma valine and isoleucine concentrations were significantly higher in the of DM-cont than in the control group, with these concentrations restored to the control level by empagliflozin treatment.

**Table 5:** Comparison of plasma amino acids concentration (nmol/mL) between control, DM-cont, and DM-empa after 12 week treatment period.

	Control (n = 12)	DM-cont (n = 16)	DM-empa (n = 5)
proline	66.8±4.2	63.6±4.3	85.6±11.4
sarcosine	2.8±0.3	3.4±0.3	4.2±0.5*
glycine	107.3±8.6	107.6±7.7	162.0±16.8*#
glutamine	662.8±49.6	466.5±61.5*	400.7±37.8
ornithine	51.9±5.9	34.6±4.2*	31.4±4.2
tryptophan	47.3±2.9	37.8±3.5	31.0±2.9*
α-aminobutyric acid	7.6±1.1	5.7±0.3*	5.0±0.1*#
alanine	271.2±18.2	326.1±18.9	246.0±6.8
valine	96.7±6.4	134.9±10.5*	84.5±10.9#
isoleucine	34.9±2.2	46.1±3.7*	28.5±3.3#

Plasma concentration of amino acids showing VIP values ≥ 1. Data were represented as mean ± SE.

* *p*<0.05 vs control.

# *p*<0.05 vs DM-cont.

## Discussion

SGLT2 inhibitors reduce blood glucose levels in an insulin-independent manner and show renoprotective effects in patients with type 2 DM, as well as in several animal models [8, 12, 21–23]. Most animal studies with SGLT2 inhibitors have been conducted in obese animals with type 2 DM [22, 24–28]. However, many type 2 DM patients, especially in Asia, are non-obese [29]. The effects of SGLT2 inhibitors on patients with renal lesions or non-obese diabetes remain unclear [29, 30]. The present study investigated the effects of empagliflozin on metabolism, renal function and histopathology in DEK rats, a novel non-obese diabetic rat model with predisposition to enlarged kidneys [14]. Compared to diabetic animal models with obese, the phenotype of diabetic DEK rat was characterized by the progressive reduction of plasma insulin concentration due to loss of pancreatic β cells, rather than insulin resistance, so that diabetic DEK rats showed decrease in BW and fat with the progression of DM. In addition, diabetic DEK rats showed increased renal parenchyma and marked enlargement of the kidneys with dilation of renal tubules [14].

SGLT2 inhibitors promote the utilization of fat, muscle and amino acids as energy sources [31–33], reducing fat mass and BW in obese humans and animals [28, 34, 35]. Loss of fat mass and decrease in weight gain have also been observed in non-obese diabetic rodents treated with SGLT2 inhibitors [31]. In addition, both humans and rodents treated with SGLT2 inhibitors showed compensatory hyperphagia to maintain their energy and nutrient balance [27, 32, 36]. In this study, empagliflozin treatment lowered blood glucose levels of DEK rats within 24 hours, with these lower concentrations maintained throughout the 12-week treatment period, similar to other studies of SGLT2 inhibitors [15, 24]. In contrast to previous findings [27, 28, 36], we found that empagliflozin treatment reduced food intake and attenuated the loss of adeps renis and the inhibition of BW gain. This unexpected result was probably associated with improvements in energy balance. Initially, urinary glucose excretion was significantly lower in DM-empa than in DM-cont rats, although the markedly higher Cglu in the DM-empa group resulted from the complete inhibition of glucose reabsorption, with FEglu being almost 100%. This finding was further supported by the significant reduction in urinary UN excretion, a parameter of protein catabolism, despite a normal BUN level, in the DM-empa group [37]. In addition, the increase in plasma TP and the decrease in urinary protein in the DM-empa group indicated that empagliflozin improved protein retention. The retention and utilization of glucose and the reversion from a catabolic to an assimilated state in rats treated with empagliflozin may have been due to its successful control of blood glucose levels.

SGLT2 inhibitors have been shown to increase urine volume, with increases in tubular reabsorption compensating for losses in body fluids and electrolytes [21, 25, 38]. Therefore, urine volume is increased immediately after the start of SGLT2 inhibitor treatment, but long-term treatment may reduce both urine volume and water intake [38, 39]. Although plasma Cre level was significantly lower in DM-cont and DM-empa than in Control group, Ccre did not differ significantly among the control, DM-cont, and DM-empa groups. Therefore, since there was no obvious evidence of glomerular hypertrophy in DEK-DM rats [14], glomerular hyperfilration might barely occurred or mild in DM-cont. In addition, enlarged kidney with increased parenchyma might have increased nephron number, indicating that enlarged kidney in both DM-cont and DM-empa might be related with reduced plasma Cre levels. Therefore, the reduced urine volume in the DM-empa group may have resulted from improvements of water reabsorption in renal tubules, not from reduced glomerular filtration rate. Overall, the increased urinary excretion of electrolytes in the DM-cont group was attenuated in the DM-empa group, indicating that the reduced plasma concentrations of electrolytes in the DM-cont group were attenuated by empagliflozin treatment via the improvement of renal tubular function. This hypothesis was supported by the consistent reductions of their clearances with decreased fraction excretions in the DM-empa group.

SGLT2 inhibitors have been reported to have deleterious effects on bone [31, 40]. Because osteoporosis is a common complication of DM, we compared BMD and BMC in the three groups of rats. BMD and BMC were significantly lower in the DM-cont than in the control group, but these reductions were attenuated in the DM-empa group. Moreover, these increases in BMD and BMC in the DM-empa group were accompanied by reductions in urinary Ca excretion. Because plasma Ca levels are strictly regulated, there were no significant differences in plasma Ca levels among the control, DM-cont, and DM-empa groups. These findings suggested that the negative Ca balance in the DM-cont group may be offset by the mobilization of Ca from bone into the circulation, with this condition being partially attenuated by the improved renal tubular function of Ca in the DM-empa group.

The attenuation of hypoproteinemia in the DM-empa group may have resulted from an improvement in proteinuria. Although glomerular hyperfiltration and hypertrophy were not clearly exhibited in the DM-cont group, the reduced blood glucose level may decrease the glomerular filtration of Alb. In addition, improvements in the tubular reabsorption of proteins and amino acids may have contributed to the synthesis of plasma proteins in the liver. Affected DEK rats are characterized by low plasma Tcho concentrations [14]. Because increased production of Alb is accompanied by increased production of Tcho in the liver [41], the higher plasma Tcho levels in the DM-empa group may be associated with the increased hepatic production of Alb. The latter may have resulted from the increased amino acid and energy supplies related to improvements in protein retention and energy balance.

Branched chain amino acid (BCAA) homeostasis is largely determined by BCAA catabolic activity in tissues [42], with defects in BCAA homeostasis regarded as an index of insulin resistance or diabetic condition [43–45]. Our GC-MS analysis of amino acids showed that the plasma concentrations of valine and isoleucine in DM-empa rats were equal to those in non-diabetic control rats, suggesting that empagliflozin treatment ameliorated protein catabolism and diabetic conditions. In addition, we observed that an empagliflozin-specific increase in plasma glycine level was associated with the improvement of diabetes. Interestingly, low plasma glycine level has been observed in human diabetes [46], and glycine administration was found to induce antioxidative and renoprotective effects in rats with streptozotocin-induced diabetes [47]. Higher plasma glycine levels may therefore be associated with the attenuations of oxidative stress and diabetic nephropathy in diabetic patients and experimental animals treated with empagliflozin. Plasma levels of amino acids are influenced by their utilization by tissues and organs throughout the body and depend on anabolic and catabolic conditions. Further studies are required to determine how changes in the levels of these and other amino acids are related to metabolic condition.

SGLT2 inhibitors exert renoprotective effects by suppressing several processes associated with kidney diseases, such as albuminuria and increased kidney weight [15, 24, 48, 49]. Renal tubule dilation and proteinuria are markers of diabetes-associated kidney damage [50]. However, empagliflozin was unable to suppress renal tubule dilation and increased urinary excretion of L-FABP in our rat model, but significantly improved tubular function. This discrepancy suggested that the empagliflozin associated improvement in tubular function resulted from minor or secondary effects not accompanied by organic alterations in renal tubules. Alternatively, because tubular dilation with injury and increased kidney weight were maintained in normoglycemic conditions in affected DEK rats, these alterations may have been genetic traits related with diabetes in DEK rats rather than secondary to hyperglycemia.

Although SGLT2 inhibitors target renal tubules, little is known about the effects of these agents on overall changes in the function of renal tubules, except that glomerular hypertrophy involves the tubular cotransport of sodium with glucose [13, 22, 24]. Studies of the effects of SGLT2 inhibitors on tubular function showed that water reabsorption was not only suppressed by osmotic diuresis but was enhanced by upregulation of aquaporin and urea transporter by an as yet undetermined mechanism [21, 25]. Renal tubular epithelial cells of diabetic patients are influenced not only by the osmotic pressure of glucose present in tubular fluid, but by excess glucose and sodium taken up into the cells via SGLT2. Recently, oxidative stress in renal tubules has been considered important for the progression of diabetic nephropathy (DN), and ipragliflozin showed renoprotective effects via preventing the overproduction of ROS in renal tubules [51]. Although no serious progression of DN was found in affected DEK rats, oxidative stress might impair the tubular function. Currently, there is no unified consensus about the effects of SGLT2 inhibitors on tubular function, suggesting a need for further studies to elucidate the mechanism by which empagliflozin improves tubular function in DEK rats.

SGLT2 inhibitors have been found to preserve β cell mass in type 1 DM and obese type 2 DM models [52, 53]. We detected β cells with weak insulin signals in the DM-empa group, but no β cells in the DM-cont group. However, OGTT showed moderate improvements in glucose tolerance without insulin secretion in the DM-empa group. Because empagliflozin has a short half-life of about 8 hours, the reduced blood glucose levels observed after a 16 hour fast and the improvement in glucose tolerance were not due to the effects of remnant empagliflozin. These findings therefore suggest that the long-term administration of empagliflozin might improve glucose metabolism and moderately reduce blood glucose level after one-shot glucose loading in an insulin-independent manner.

In conclusion, the results of the present study indicate that empagliflozin ameliorates systemic metabolism and renal tubular function in DEK rats, although the detailed mechanisms underlying the unique response of these animals to empagliflozin remain unclear. These findings also indicate that DEK rats are a useful model for studying and evaluating the multiple biological effects of SGLT2 inhibitors in diabetes.

## Supporting information

S1 Fig Effects of empagliflozin on food and water intake and urinary volume. Food intake (A), water intake (B) and urinary volume (C) over 24 h of rats in the control (n = 3), DM-cont (n = 3) and DM-empa (n = 5) groups during week 12. All data were expressed as absolute values. * *p*<0.05.
(TIF)

S2 Fig PLS-DA score plots, loading plots and variables important for prediction (VIP) values of plasma amino acids. **(A)** PLS-DA score plots showing separations among rats in the control (n = 12, blue circles), DM-cont (n = 16, red squares), and DM-empa (n = 5, green triangles) groups. The model was validated by the values of R2Y (cum 0.512) and Q2Y (cum 0.434). (B) PLS-DA loading plots of all amino acids detected in our assay. (C) VIP values of the amino acids. VIP values ≥1 were considered statistically significant (red bars).
(TIF)
