# Supplementary figures and images for: Empagliflozin ameliorates symptoms of diabetes and renal tubular dysfunction in a rat model of diabetes with enlarged kidney (DEK)
Source: PLoS One. 2021 May 4;16(5):e0251135. doi: 10.1371/journal.pone.0251135 (PMC8096081; doi:10.1371/journal.pone.0251135)

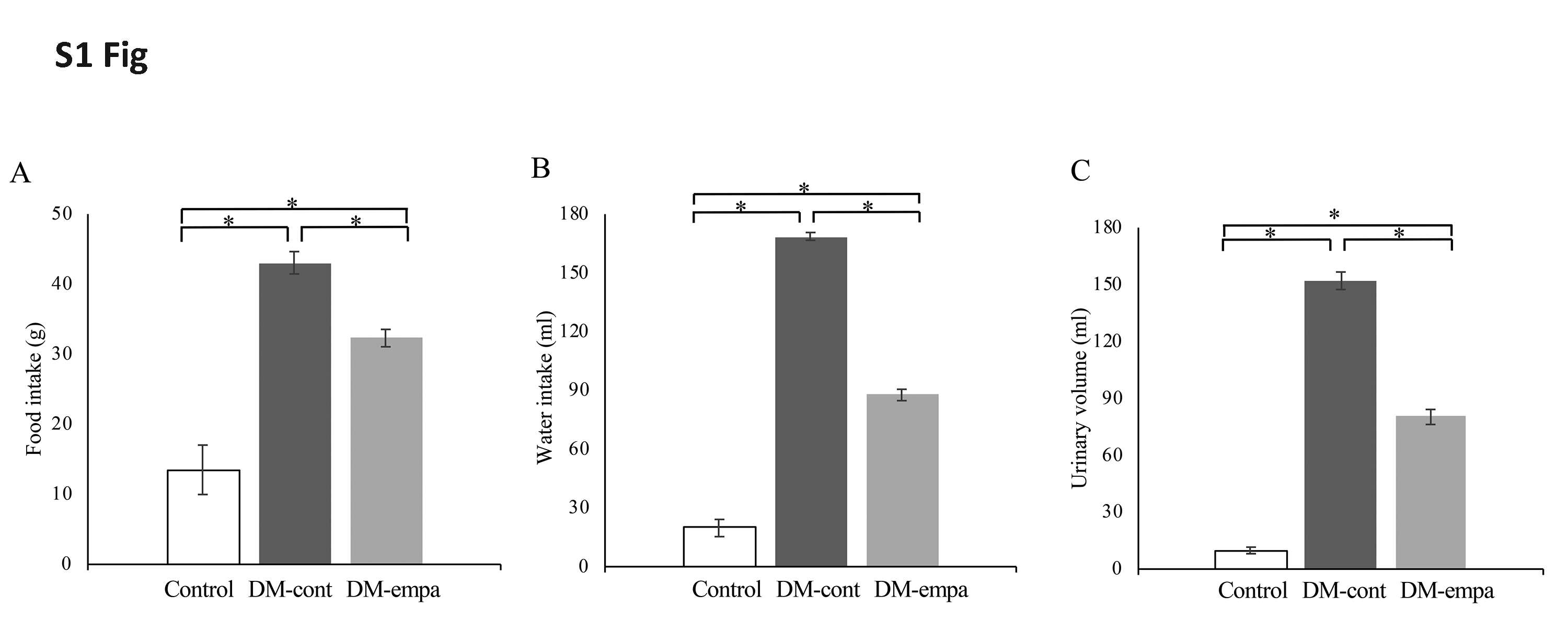

Supplement: S1 Fig — Food intake (A), water intake (B) and urinary volume (C) over 24 h of rats in the control (n = 3), DM-cont (n = 3) and DM-empa (n = 5) groups during week 12. All data were expressed as absolute values. * p<0.05. (TIF) [file pone.0251135.s001.tif]

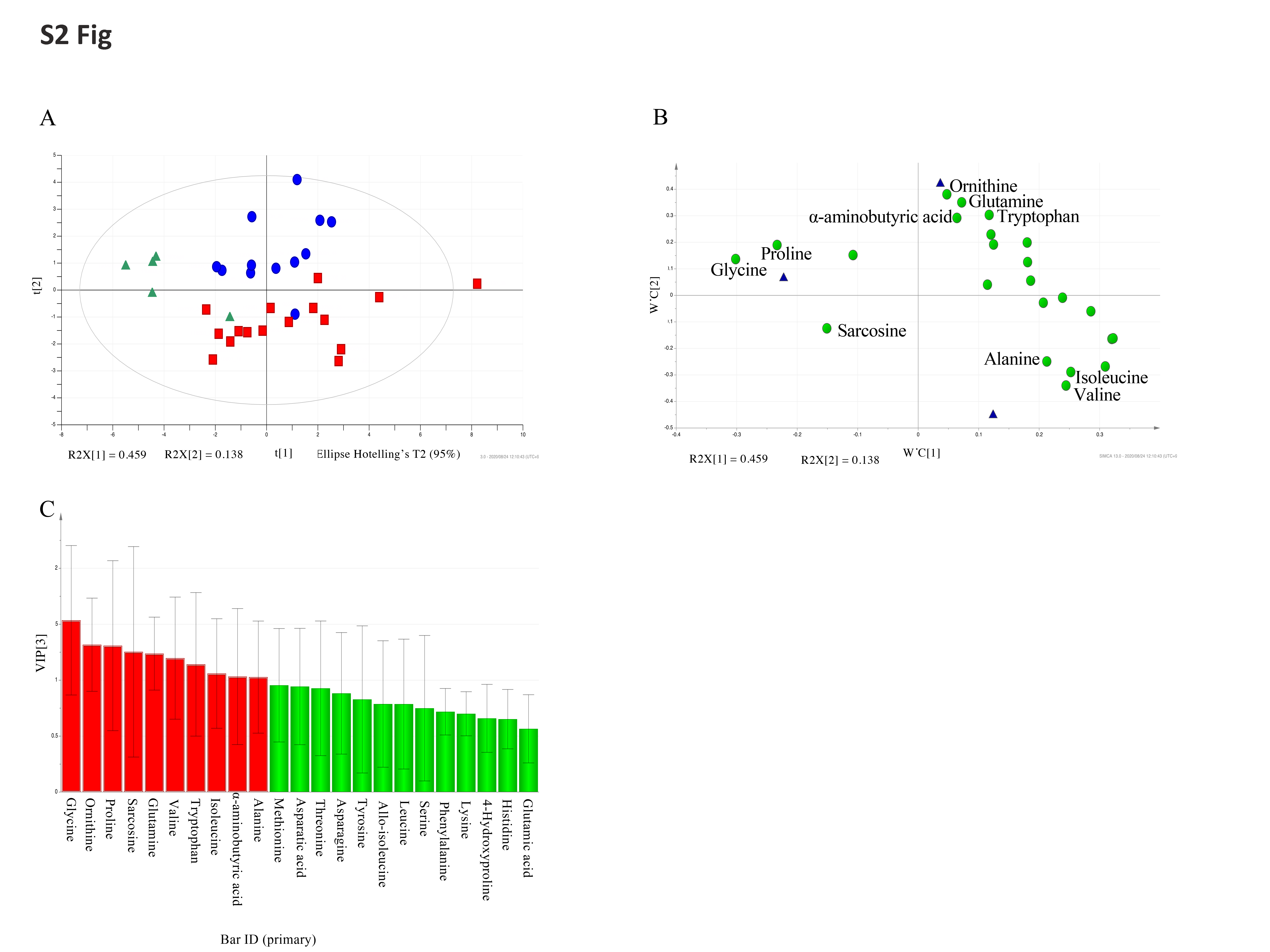

Supplement: S2 Fig — (A) PLS-DA score plots showing separations among rats in the control (n = 12, blue circles), DM-cont (n = 16, red squares), and DM-empa (n = 5, green triangles) groups. The model was validated by the values of R2Y (cum 0.512) and Q2Y (cum 0.434). (B) PLS-DA loading plots of all amino acids detected in our assay. (C) VIP values of the amino acids. VIP values ≥1 were considered statistically significant (red bars). (TIF) [file pone.0251135.s002.tif]
